# Hsp70 Regulates Immune Response in Experimental Autoimmune Encephalomyelitis

**DOI:** 10.1371/journal.pone.0105737

**Published:** 2014-08-25

**Authors:** M. José Mansilla, Carme Costa, Herena Eixarch, Vanja Tepavcevic, Mireia Castillo, Roland Martin, Catherine Lubetzki, Marie-Stéphane Aigrot, Xavier Montalban, Carmen Espejo

**Affiliations:** 1 Servei de Neurologia-Neuroimmunologia, Centre d′Esclerosi Múltiple de Catalunya (Cemcat), Vall d′Hebron Institut de Recerca (VHIR), Hospital Universitari Vall d′Hebron, Barcelona, Spain; 2 Universitat Autònoma de Barcelona, Bellaterra, Cerdanyola del Vallès, Barcelona, Spain; 3 Sorbonne Universités UPMC Univ Paris 06, UM-75, ICM-GH Pitié-Salpêtrière, Paris, France, Inserm, U1127, Paris, France, CNRS, UMR 7225, Paris, France; 4 Department of Neuroimmunology and Multiple Sclerosis Research, Neurology Clinic, University Hospital Zürich, Zürich, Switzerland; Hospital Nacional de Parapléjicos – SESCAM, Spain

## Abstract

Heat shock protein (Hsp)70 is one of the most important stress-inducible proteins. Intracellular Hsp70 not only mediates chaperone-cytoprotective functions but can also block multiple steps in the apoptosis pathway. In addition, Hsp70 is actively released into the extracellular milieu, thereby promoting innate and adaptive immune responses. Thus, Hsp70 may be a critical molecule in multiple sclerosis (MS) pathogenesis and a potential target in this disease due to its immunological and cytoprotective functions. To investigate the role of Hsp70 in MS pathogenesis, we examined its immune and cytoprotective roles using both *in vitro* and *in vivo* experimental procedures. We found that Hsp70.1-deficient mice were more resistant to developing experimental autoimmune encephalomyelitis (EAE) compared with their wild-type (WT) littermates, suggesting that Hsp70.1 plays a critical role in promoting an effective myelin oligodendrocyte glycoprotein (MOG)-specific T cell response. Conversely, Hsp70.1-deficient mice that developed EAE showed an increased level of autoreactive T cells to achieve the same production of cytokines compared with the WT mice. Although a neuroprotective role of HSP70 has been suggested, Hsp70.1-deficient mice that developed EAE did not exhibit increased demyelination compared with the control mice. Accordingly, Hsp70 deficiency did not influence the vulnerability to apoptosis of oligodendrocyte precursor cells (OPCs) in culture. Thus, the immunological role of Hsp70 may be relevant in EAE, and specific therapies down-regulating Hsp70 expression may be a promising approach to reduce the early autoimmune response in MS patients.

## Introduction

Heat shock proteins (HSPs) are widely known as conserved cytoprotective proteins due to their function as chaperones, in which they help to properly fold newly synthesised proteins, prevent protein aggregation and degrade unstable and misfolded proteins [1]–[5]. Under stressful conditions, the expression of specific HSPs is induced to control cellular damage and restore cellular homeostasis [6]–[8]. In the HSP70 family, Hsp70 (encoded by the Hsp72, Hsp70.1 and Hsp70.3 genes) is one of the most important stress-inducible proteins [9]. Intracellular Hsp70 not only mediates chaperone-cytoprotective functions but also can block multiple steps in the apoptosis pathway [10]–[12]. In addition, Hsp70 is actively released into the extracellular milieu where it can act as cytokine and peptide adjuvant, thereby promoting both the innate and adaptive immune responses [13]–[16].

Multiple sclerosis (MS) is an autoimmune disorder in which activated CD4+ T cells initiate an inflammatory response in the central nervous system (CNS). This activation results in inflammation, gliosis, demyelination as well as oligodendrocyte and neuronal loss [17]. Accordingly, immunomodulation and cytoprotection of specific cell populations in the CNS have been established as two key aspects of MS therapeutics. Nevertheless, MS treatments have mainly focused on controlling the immune response due to the lack of effective neuroprotective treatments.

Previous studies have indicated that Hsp70 is a critical molecule in MS pathogenesis [18] and a potential disease target due to its immunological and cytoprotective functions.

The inflammatory and oxidative environment taking place in the CNS of MS patients and in the experimental autoimmune encephalomyelitis (EAE) animal model induces the overexpression of most of HSP, including Hsp70 [19]–[23]. This inflammatory-Hsp70 induction occurs predominantly in oligodendrocytes [24] and, as in other experimental models of neuropathological diseases such as Alzheimer's and Parkinson's diseases [25], [26], Hsp70 overexpression in the CNS of MS patients and EAE animals suggests a neuroprotective role for this protein. By contrast, because Hsp70 can act as an adjuvant Hsp70–myelin basic protein (MBP) and Hsp70–proteolipid protein (PLP) complexes have been found in MS lesions, which have been considered highly immunogenic [19], [27], [28].

Recently, our group has shown a baseline increase in *HSPA1A* gene expression in peripheral blood mononuclear cells (PBMCs) obtained from MS patients compared with those of healthy donors (HDs). Gene expression analyses were confirmed by Hsp70.1 protein expression in both T lymphocytes (CD4+ and CD8+) and monocytes from MS patients under basal conditions, which could reflect immunological activation in MS patients. Our data suggested altered immune responses in MS and may indicate a state of chronic stress or increased vulnerability to physiological immune responses in MS patients [29].

In this study, we aimed to investigate the role of Hsp70 in MS pathogenesis using both *in vitro* and *in vivo* experimental procedures. *In vitro* studies showed that subjecting mixed CNS cultures to inflammatory stress resulted into oligodendrocyte precursor cells (OPCs) apoptosis. However, this OPC vulnerability was not influenced by Hsp70 deficiency. In the EAE model we demonstrated that Hsp70 is one of the molecules that promote an efficient antigen-specific immune response required to develop EAE, but it is not crucial to protect CNS cells from the inflammatory damage. Thus, Hsp70.1 inhibition/down-regulation may be an effective therapeutic strategy to reduce the early autoimmune response in MS patients.

## Materials and Methods

### Mice

C57BL/6J Hsp70.1 knock-out (KO) mice [30] and age- and sex-matched WT littermates were purchased from Macrogen (Seoul, Korea) and used to establish mouse colonies that were bred in our animal facility at Vall d′Hebron Research Institute. Seven-week-old female C57BL/6J mice were purchased from Harlan Laboratories (Italy) for the Hsp70.1 knock-down studies. The mice were housed under standard light- and climate-controlled conditions, and standard chow and water were provided ad libitum. All experiments were performed in strict accordance with EU and governmental regulations (Generalitat de Catalunya Decret 214/97 30th July). The Ethics Committee on Animal Experimentation of the Vall d′Hebron Research Institute approved all procedures described in this study (protocol number: 11/06 CEEA). Measures to improve welfare assistance and clinical status as well as endpoint criteria were established to minimise suffering and ensure animal welfare. Briefly, wet food pellets are placed on the bed-cage when the animals begin to develop clinical signs to facilitate access to food and hydration. If weight loss was greater than 15%, mice received subcutaneously 0.5 ml of 10% glucose. Mice suffering severe disease (score 5) or 30% weight loss were euthanized in accordance with our governmental ethical guidelines.

### EAE induction and follow-up analyses

Anaesthetised mice were immunised with subcutaneous injections of 200 µl of phosphate buffered saline (PBS) containing 200 µg of 35–55 myelin oligodendrocyte glycoprotein peptide (MOG_35–55_) (Proteomics Section, Universitat Pompeu Fabra, Barcelona, Spain) emulsified in complete Freund's Adjuvant (CFA) (Sigma Chemical, St. Louis, MO, USA) containing 4 mg/ml *Mycobacterium tuberculosis* H37RA (Difco Laboratories, Detroit, MI, USA). At day 0 and 2 post-immunisation (p.i.), the mice received 250 ng of pertussis toxin intravenously (Sigma Chemical). A total of 51 Hsp70.1 KO and 36 WT mice were immunised in three independent experiments. A group of 3–4 animals per group were used as control mice, which were immunised in the same manner using PBS in the absence of the peptide. All animals were weighed and examined daily for welfare and clinical status as well as neurological signs according to the following criteria: 0, no clinical signs; 0.5, partial loss of tail tonus for at least two consecutive days; 1, paralysis of the whole tail; 2, paresis of one or both hind limbs; 3, hind limb paraplegia; 4, tetraparesis; 5, tetraplegia; and 6, death [31]. Clinical follow-up analyses were performed in a blinded manner by two observers. All data presented are in accordance with the guidelines suggested for EAE publication [32].

### *In vivo* siRNA administration

The C57BL/6J WT mice were immunised as previously described. At day 7 p.i., once the first EAE clinical symptoms were detected, the animals were randomised into two groups (n = 5 mice in each group) and then were intravenously injected with either 200 µl of PBS containing 2 mg/kg of a pool of four specific mouse Hsp70.1 (HSPA1B, ref. E-065710) siRNAs or non-targeting siRNAs (control, ref. D-001910) (accell SMART pool siRNA, Dharmacon, Thermo Fisher Scientific, Waltham, MA, USA). At day 14 p.i., when the disease had already been established, the mice received a second dose of Hsp70.1 or non-targeting siRNA. The administration of the siRNAs was performed in a blinded manner.

### Splenocyte proliferation and cytokine production

At day 12 p.i. and at the end of the experiment (day 29 p.i.), a subgroup of Hsp70.1 KO and WT mice (day 12pi: n = 4 in each group and day 29pi: WT n = 3, Hsp70.1 KO n = 5) were euthanized using carbon dioxide (>70%), and the spleens were removed. Spleen cell suspensions were prepared by grinding the spleens through a wire mesh, and the resulting cells were cultured in 96-well plates at 2×10^5^ cells/well in a total volume of 200 µl of Iscove's modified Dulbecco's medium (IMDM) (PAA Laboratories GmbH, Pasching, Austria) supplemented with 10% HyClone® FetalClone I (Thermo Fisher Scientific, Waltham, MA, USA), 50 µM 2-mercaptoethanol (Sigma Chemical), 2 mM glutamine, 50 U/ml penicillin and 50 mg/ml streptomycin, all of which were obtained from Gibco BRL (Paisley, UK). The cultures (five replicas for each condition) were stimulated with 5 µg/ml MOG_35–55_. The supernatants (50 µl/well) were harvested after 48 h of culture and stored to further assess the cytokine release. Next, 1 µCi/well of [^3^H]-thymidine (PerkinElmer, Waltham, MA, USA) was added to each well. The cultures were maintained under the same conditions for an additional 18 h, and the levels of incorporated radioactivity were determined using a scintillation counter (Wallac, Turku, Finland). The stimulation index (SI) values for each sample were calculated as the mean counts per minute (cpm) of the five replicas of MOG_35–55_-stimulated cultures divided by the mean cpm of the five replicas from the non-stimulated cultures. The cytokine secretion was determined in the culture supernatants using the FlowCytomix Th1/Th2/Th17 10 plex kit (Bender MedSystems Inc., Burlingame, CA) and FacsCanto cytometer (Becton Dickinson) according to the manufacturer's instructions.

### Primary antibodies

For the histopathological studies, the following primary antibodies and dilutions were used: anti-CD45 (pan-leukocyte marker) (rat IgG2b, 1∶300, R&D Systems, Abingdon, OX, UK), anti-CD3 (T lymphocytes) (rabbit polyclonal, 1∶100, Dako, Glostrup, Denmark), anti-GFAP (astrocytes) (rabbit polyclonal, 1∶500, Dako), *Lycopersicon esculentum* agglutinin (LEA, microglia/macrophages) (1∶100, Sigma), anti-neurofilament H non-phosphorylated (SMI32, axonal damage) (mouse IgG, 1∶100, Covance Inc.), anti-NG2 (oligodendrocyte progenitor cells) (rabbit polyclonal, 1∶200, Merck Millipore, Billerica, MA, USA), anti-malondialdehyde (MDA, lipid-oxidation) (rabbit polyclonal, 1∶100, Alpha Diagnostic Int., San Antonio, TX, USA), anti-nitrotyrosine (NITT, protein-oxidation) (rabbit polyclonal, 1∶100, Alpha Diagnostic Int.), anti-heme oxygenase 1 (HOx, oxidative stress) (rabbit polyclonal, 1∶100, Enzo Life Sciences, Lausen CH, Switzerland) and anti-inducible nitric oxide synthase (iNOS2, oxidative stress) (rabbit polyclonal, 1∶100, Santa Cruz Biotechnologies, Dallas, TX, USA).

For immunostaining of the CNS cultures, the following primary antibodies were used: anti-O4 (OPC) (mouse IgM, 1∶5, hybridoma generously donated by Dr. Sommer), anti-myelin basic protein (MBP, mature oligodendrocytes) (chicken IgY, 1∶200, Millipore), anti-cleaved Caspase 3 (Casp3) (rabbit polyclonal, 1∶500, Cell Signaling Technologies, Danvers, MA, USA), anti-NeuN (neurons) (mouse IgG1, 1∶300, Merck Millipore), anti-GFAP (astrocytes) (rabbit polyclonal, 1∶500, Dako), isolectin B4 conjugated to fluorescein-isothiocyanate (FITC) (mouse microglial cells) (1∶1000, Sigma), anti-CD11b/c (rat microglial cells) (mouse IgG2a, 1∶100, BD Pharmingen, San Diego, CA) and anti-CD68 (activated rat microglial cells) (mouse IgG1 1∶200, AbD Serotec, UK).

### Histopathology

For the histopathological studies, Hsp70.1 KO and WT mice were euthanised at day 12 p.i. (WT: n = 3 and Hsp70.1 KO: n = 6) or at the end of the experiment, day 29 p.i. (WT: n = 7 and Hsp70.1 KO: n = 13). The mice treated with Hsp70.1 (n = 5) or non-targeting siRNAs (n = 5) were euthanised at the end of the experiment. The brains and spinal cords were removed, fixed with 4% paraformaldehyde and embedded in paraffin wax. Four-micrometre-thick tissue sections were stained with hematoxylin and eosin (HE) and Klüver-Barrera (KB) to assess the degree of inflammation and demyelination, respectively. Immunostaining was also performed. Briefly, endogenous peroxidase activity was blocked by incubating the tissue sections in 2% hydrogen peroxide, 70% methanol and PBS for 20 min. Antigen unmasking was performed in 10 mM citrate (pH  =  6) for anti-CD45, SMI32, NG2, NITT, HOx and iNOs or protease type XIV (Sigma Chemical) for anti-CD3 antibody. Non-specific protein binding was blocked using 2% bovine albumin in PBS (blocking solution) at room temperature for 1 h. The sections were incubated overnight at 4°C using the primary antibodies detailed in the Primary antibodies section. Next, all of the samples were incubated at room temperature for 1 h with biotinylated anti-rabbit, anti-rat or anti-mouse IgG (all from DakoCytomation) (1∶200 dilution in blocking solution) secondary antibodies. Finally, the avidin–biotin-peroxidase complex (ImmunoPure ABC Peroxidase Staining Kits, Pierce, IL, USA), which was diluted 1∶100 in PBS, was added for 1 h at room temperature. The peroxidase reaction was visualised with 2.5 mg/ml 3,3′-diaminobenzidine and 0.05% hydrogen peroxide. The primary antibodies were omitted in the negative controls. No signal was observed in any of the control slides.

Cell infiltration was evaluated using HE staining according to the following criteria: 0, no lesion; 1, cellular infiltration only in the meninges; 2, very discrete and superficial infiltrates in the parenchyma; 3, moderate infiltration (less than 25%) in the white matter; 4, severe infiltration (less than 50%) in the white matter; and 5, more severe infiltration (greater than 50%) in the white matter. Demyelination (KB staining) was scored as follows: 0, no demyelination; 1, little demyelination, only around the infiltrates and involving less than 25% of the white matter; 2, demyelination involving less than 50% of the white matter; and 3, diffuse and widespread demyelination involving more than 50% of the white matter. Quantification of the stained cells was performed in three matched areas (0.25 mm^2^) along the spinal cord. The HE and KB staining and all immunostaining markers were evaluated in a blinded manner.

### CNS cell cultures

Brains were obtained from 1- to 2-day-old postnatal Hsp70.1 KO or WT mice and dissected in Hanks buffer (Hanks 10x, Gibco), supplemented with 0.01 M HEPES buffer, 0.75% sodium bicarbonate (Gibco) and 1% penicillin/streptomycin. A pool of 3-5 WT or Hsp70.1 KO dissected brains was enzymatically dissociated with papain (30 µg/ml in DMEM with 0.24 µg/ml L-cysteine and 40 µg/ml DNase I) and then mechanically dissociated using 150 µm and 65 µm filters. To obtain CNS preparation enriched in oligodendrocyte lineage cells, a percoll density gradient was performed as previously described [33] to obtain the fraction of cells enriched in oligodendroglial precursor cells. The cells were resuspended in modified Bottenstein Sato medium (DMEM containing 0.5% foetal calf serum (FCS), 2 mM L-glutamine, 10 µM insulin, 5 ng/ml sodium selenite, 100 µg/ml transferrin, 0.28 µg/ml albumin, 60 ng/ml progesterone, 16 µg/ml putrescine, 40 ng/ml triiodothyronine and 30 ng/ml L-thyroxine), and 40,000 cells per well were seeded onto poly-L-lysine-coated (40 µg/ml, Sigma) glass coverslips in 24-well plates.

### Preparation of microglial cells

Rat microglial cells were obtained from Wistar neonatal glial mix cultures. Brains were dissected and dissociated using the papain and mechanical procedure (as indicated above). After filtration, cells were cultured in DMEM containing 10% FCS in T125 flasks coated with polyethylenimine. At 10 days in vitro, the flasks were subjected to 1 h of shaking at 180 rpm. This procedure detaches preferentially microglial cells that were then washed, counted, and used for induction of inflammatory stress in mixed CNS cultures.

### Induction of inflammation in mixed CNS cell cultures

The CNS cell cultures were challenged with an inflammatory stress stimulus for 24 h. The inflammatory stimulus consisted on the replacement of 50% of the cultured medium by culture medium containing 20,000 or 16,000 microglial cells and different concentrations of LPS and IFN-γ (10 µg/ml LPS plus 150 ng/ml IFN-γ, 1 µg/ml LPS plus 100 ng/ml IFN-γ or 100 ng/ml LPS plus 100 ng/ml IFN-γ). CD11b/c and CD68 staining was performed to confirm microglial activation. Cells co-cultured with the same amount of microglial cells without LPS and IFN-γ stimulus were used as control CNS cultures.

### Immunocytochemistry and detection of apoptosis

Following 24 h of inflammatory or non-stimulated culture conditions, the CNS cells were stained with anti-O4 (marker for late OPCs), anti-MBP (marker for mature oligodendrocytes), anti-NeuN (marker for neurons), and anti-Casp3 (marker for apoptotic cells) antibodies (see the Primary antibodies section). The cells were washed and fixed with 4% paraformaldehyde for 15 min at room temperature. After washing, the cells were incubated in blocking solution (0.1% Triton X100, 10% horse serum) for 20 min at room temperature. The samples were incubated with the primary antibodies overnight at 4°C or for 1 h at room temperature in blocking solution. After several washes, Alexa-488- or Alexa-568-conjugated-secondary antibodies were incubated for 90 min at room temperature. Secondary antibodies obtained from Molecular Probes (Invitrogen, Cergy, Pontoise, France) were used at a 1:1000 dilution. The cell nuclei were stained with Hoechst solution (1 µg/ml), and the immunocytochemistry preparations were mounted in Fluoromount-G (Clinisciences, France). For the O4 staining, *in vivo* immunocytochemistry was performed by incubation with the anti-O4 antibody for 30 min at 37°C in DMEM containing 10% horse serum followed by fixation of the cells with 4% paraformaldehyde. Negative controls were performed by omitting the primary antibodies. The stainings were observed by using a fluorescence microscope Zeiss Imager.Z1. Images were acquired with an AxioCam camera equipped with an ApoTome module and analysed using ImageJ software. The percentage of Casp3+ cells from each culture was calculated as the sum of the number of positive cells obtained from three representative microscope fields divided by the sum of the total number of cells (cells positive for Hoechst staining). The percentage of O4+Casp3+, MBP+Casp3+, and NeuN+Casp3+ cells was calculated as the sum of double positive cells divided by the sum of the total number of each population (O4+, MBP+, and NeuN+ cells). To determine the effect of the inflammatory stimulus, the fold change values of each population was calculated in WT and Hsp70.1 KO mice with respect to their matched non-stimulated control cultures.

### Statistical analysis

The data were expressed as the mean ± standard deviation (SD) values unless otherwise stated. Statistical analyses were performed using SPSS 17.0 (SPSS Inc, Chicago, IL, USA) for MS-Windows and Graphpad Prism 5. The Mann-Whitney test was applied to compare the mean values between groups; however, for large samples (n≥30) and very small samples (n≤3), an unpaired t-test was performed [34]. A paired t-test was used to analyse the effect of the inflammatory stress on the cell cultures. Fisher's exact test was used to compare qualitative variables. Differences were considered statistically significant when p<0.05.

## Results

### Reduced EAE susceptibility in Hsp70.1-deficient mice

**Figure 1:**
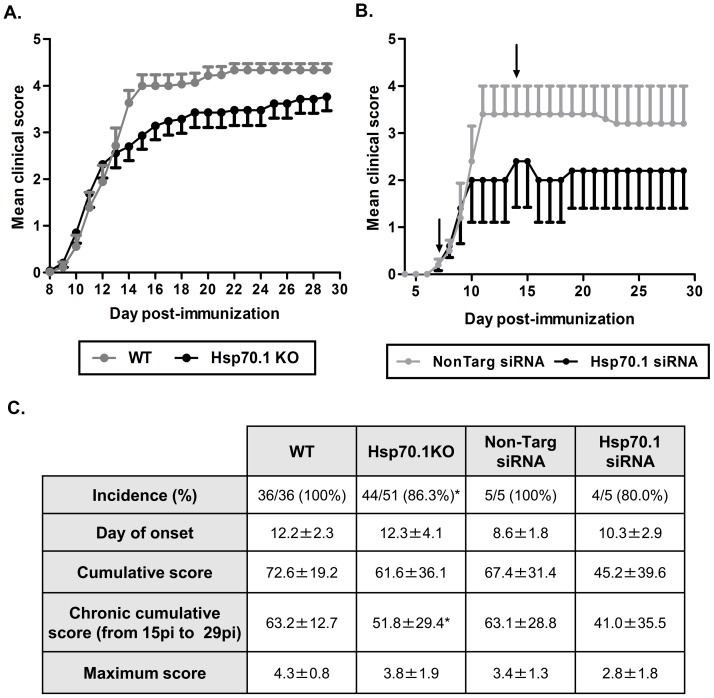
Role of Hsp70 in EAE development and clinical course. A. Hsp70.1 knock-out (Hsp70 KO) mice showed a reduced susceptibility to EAE compared with the wild-type (WT) mice after immunisation with the MOG_35–55_ peptide (86.3% *vs*. 100%, p = 0.038; n =  51 and n = 36, respectively) resulting in less disease severity in the Hsp70.1 KO mice compared with the WT mice during the chronic phase of EAE (p = 0.029). B. When Hsp70.1 expression was down-regulated using the systemic administration of siRNA on days 7 and 14 after immunisation, the mice developed a milder clinical course compared with the mice administered non-targeting siRNA (n = 5 in each group). The arrows indicate when the siRNA was administered. The data were expressed as the mean of the clinical score. The error bars correspond to the standard error of the mean (SEM). C. Summary of the EAE clinical data in WT and Hsp70.1 KO mice and in WT mice treated with Hsp70.1 or non-targeting siRNA. The data were expressed as the mean±SD. *p<0.05.

To study the role of the inducible Hsp70 protein in EAE pathogenesis, we immunised a total of 51 Hsp70.1 KO mice and 36 WT littermates with MOG_35–55_, in three independent experiments. Mice developed a chronic non-remitting EAE clinical course. We observed a reduction in the incidence of EAE in the Hsp70.1 KO mice compared with their WT littermates (86.3% *vs*. 100%, p = 0.038). Because WT mice were 100% incident and remaining without clinical symptoms is the best improvement of the disease, EAE incident and non-incident mice were included in the statistical analysis. The severity of the disease in the Hsp70.1 KO mice was reduced during the chronic phase of EAE (from day 15 p.i. to day 29 p.i.), being the chronic cumulative clinical score reduced in the Hsp70.1 KO mice compared with the WT mice (51.79±29.40 *vs*. 63.22±12.70, p = 0.029) (Fig. 1A and C).

To guarantee that Hsp70.1 KO mice do not have any compensatory mechanisms to supply Hsp70 lacking that could mask its real role in EAE, C57BL/6J WT mice were immunised with MOG_35–55_. At day 7 p.i., once the first EAE clinical symptoms were detected, the animals were randomised into two groups and then intravenously injected with Hsp70.1-specific siRNA or non-targeting siRNA (n = 5 in each group, 3 mice with clinical score = 0 and 2 mice with clinical score = 0.5 in both groups). At day 14 p.i., when the disease had already been established, the mice received a second dose of Hsp70.1 or non-targeting siRNA. Consistent with previous results, Hsp70.1 knock-down mice showed milder EAE progression compared to control mice administrated with non-targeting siRNA, as before EAE incident and non-incident mice were included in the statistical analysis (Fig. 1B and C).

### Increased MOG-specific proliferative response in Hsp70.1-deficient mice

**Figure 2:**
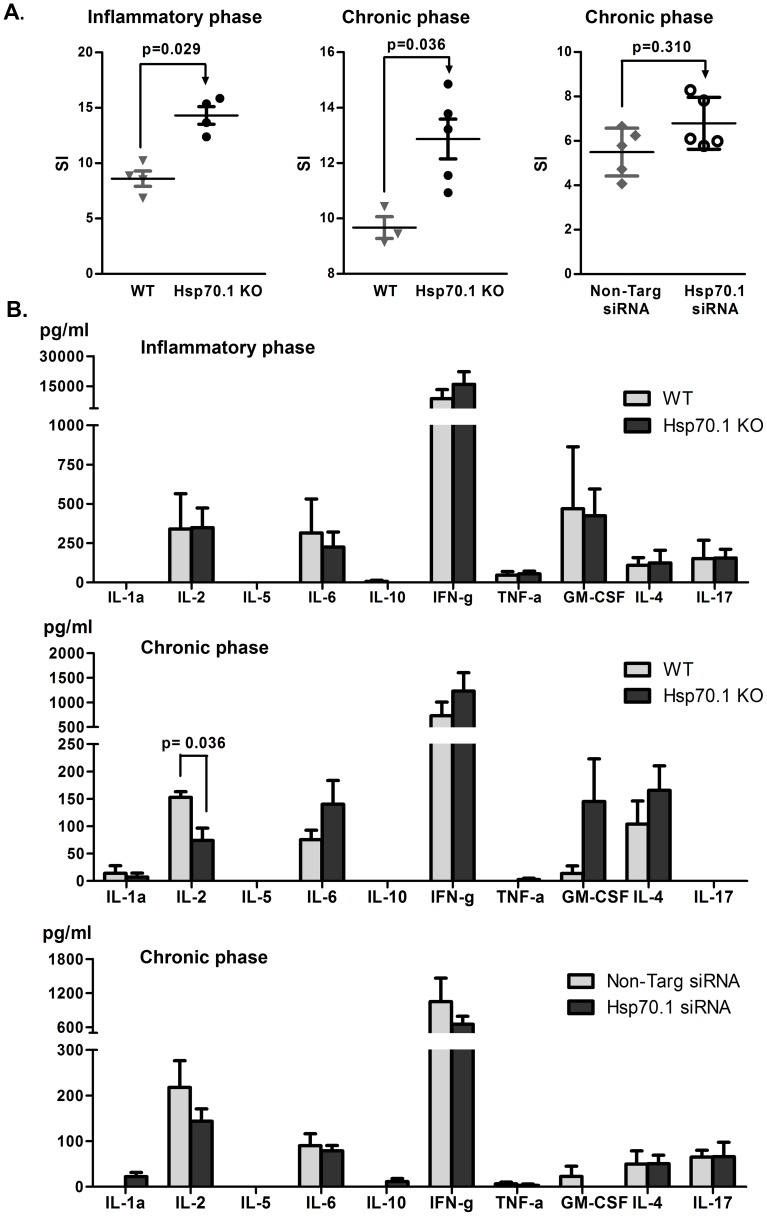
Increased MOG-proliferative response in the splenocytes of Hsp70.1-deficient mice. A. MOG-induced proliferative response of Hsp70.1-deficient and control splenocytes. The data were expressed as the mean of the stimulatory indices (SI). The error bars correspond to the SEM. Splenocytes from WT (▾) and Hsp70.1 KO (•) mice were obtained during the inflammatory phase [day 12 p.i., (left graph), n = 4 in each group] and chronic phase of EAE [day 29 p.i. (middle graph), WT: n = 3 and KO: n = 5]. Hsp70.1 KO splenocytes showed increased MOG-specific proliferation at day 12 p.i. (p =  0.029) and at day 29 p.i. (p =  0.036) compared with the WT splenocytes. The MOG-specific proliferation of splenocytes of non-targeting (♦) or Hsp70.1 (○) siRNA-treated mice (right graph) was assessed at day 29 p.i. (n = 5 in each group). B. Determination of the cytokine Th1/Th2/Th17 profile secretion following MOG-specific stimulation of splenocytes from Hsp70.1-deficient (black columns) and WT (grey columns). The increased cell proliferation of Hsp70.1-deficient splenocytes following antigen-specific stimulation was not related to an increase in cytokine production. A reduced IL2 level was only detected at day 29 p.i. in the Hsp70.1 KO splenocytes, probably due to the increased proliferative splenocyte response observed in these mice. The error bars represent the SEM.

To determine the role of inducible Hsp70 in the immune response, we investigated the proliferative capacity of Hsp70.1 KO splenocytes. Spleens were removed at day 12 p.i. (inflammatory phase) and at day 29 p.i. (chronic phase). Hsp70 deficiency strongly enhanced the *in vitro* MOG-specific splenocyte proliferation compared with the WT cells in both the inflammatory (14.31±1.60 *vs*. 8.60±1.39 p = 0.029) and chronic (12.87±1.61 *vs*. 9.67±0.68 p = 0.036) phases of the disease (Fig. 2A). Similarly, WT mice administered with Hsp70.1-specific siRNA exhibited an increased but not significant (p = 0.310) MOG-specific proliferative response in the chronic phase compared with the mice administered with non-targeting siRNAs, although these differences were not significant (Fig. 2A).

### Hsp70.1 deficiency did not alter the cytokine profile

To study whether the differential proliferative T cell response between the Hsp70.1-deficient and control mice was associated with a specific Th profile, cytokine release was analysed during the inflammatory (day 12 p.i.) and chronic (day 29 p.i.) phases of the disease. At day 12 p.i., we did not find any difference in the examined cytokines potentially involved in the increase of the MOG-specific cell proliferative response in Hsp70.1 KO mice. At day 29 p.i., only a reduced production of IL-2 was detected in the Hsp70.1 KO splenocytes, probably related with its consumption due to the increased proliferative cell response observed in Hsp70.1 KO mice. No differences were found between mice treated with either Hsp70.1 or non-targeting siRNA (Fig. 2B).

### Similar neuropathological alterations in Hsp70.1-deficient and control mice

**Figure 3:**
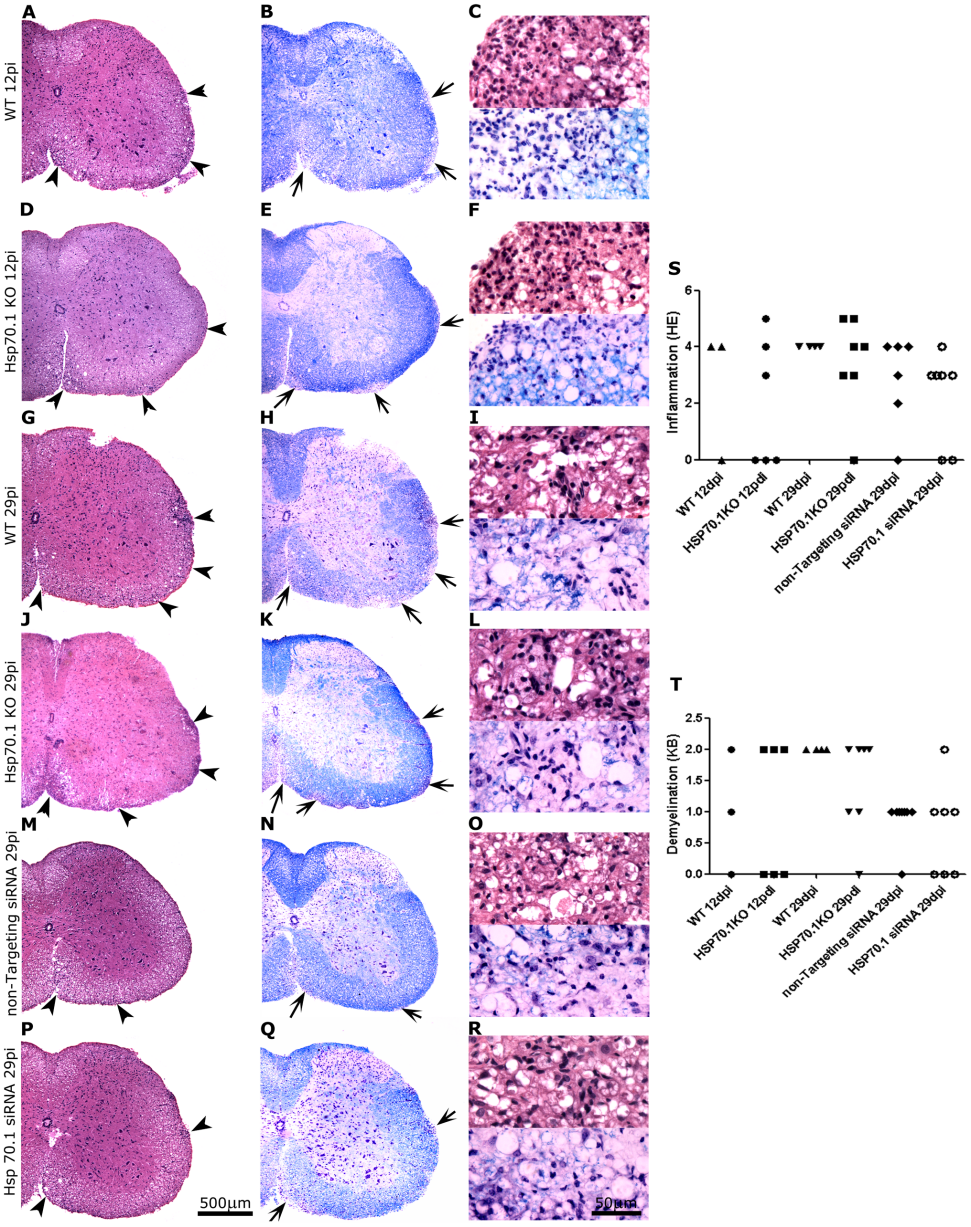
Histopathological study of the spinal cords of Hsp70.1 KO, WT and siRNA-treated mice. HE staining (A, D, G, J, M, P) shows inflammatory infiltration (arrow heads), and KB staining (B, E, H, K, N, Q) demonstrates demyelination (arrows) in the white matter of the spinal cord. Detail of inflammatory infiltration and demyelination are shown. Similar levels of inflammation (A, D) and demyelination (B, E) were observed in the WT and Hsp70.1 KO mice at day 12 p.i, when neutrophils and lymphocytes were detected and scanty demyelination was observed (C, F). At day 29 p.i, WT and Hsp70.1 KO mice had similar degree of inflammation (G, J), mainly composed by lymphocytes and macrophages (I, L), with evident demyelination (H, K, I, L). No differences were observed in inflammation (M, P, O, R) and demyelination (N, Q, O, R) in the mice treated with non-targeting siRNA compared with those treated with Hsp70.1 siRNA. Representative mice with a clinical score of 4 are shown in the figure. Graphs represent qualitative inflammatory (S) and demyelination (T) scores, assessed by HE and KB respectively, for WT and Hsp70.1 KO mice at day 12 p.i. and 29 p.i., and also for mice treated with either non-targeting or Hsp70.1 siRNA at day 29 p.i.

A histopathological study of Hsp70.1 KO and WT mice during the inflammatory phase of EAE (day 12 p.i.) revealed the presence of inflammatory infiltrates (consisting of neutrophiles, lymphocytes and a few macrophages) in the white matter of the spinal cord and subarachnoidal space of the brain, with discrete demyelination of the brain stem. In the spinal cord, the inflammatory infiltration (mainly consisting of macrophages and lymphocytes) was higher and the demyelination was more extensive during the chronic phase of the disease (day 29 p.i.) than during the inflammatory phase of EAE. However, no differences were observed with regards to inflammation or demyelination between the Hsp70.1 KO and WT mice at any phase of the disease (Fig. 3). As expected, the Hsp70.1 KO mice that did not develop EAE did not demonstrate inflammatory infiltration or demyelination in the CNS (data not shown). Otherwise, the EAE mice with matched clinical scores administered with either Hsp70.1 or non-targeting siRNAs did not exhibit any differences in either inflammatory infiltration or demyelination (Fig. 3).

**Figure 4:**
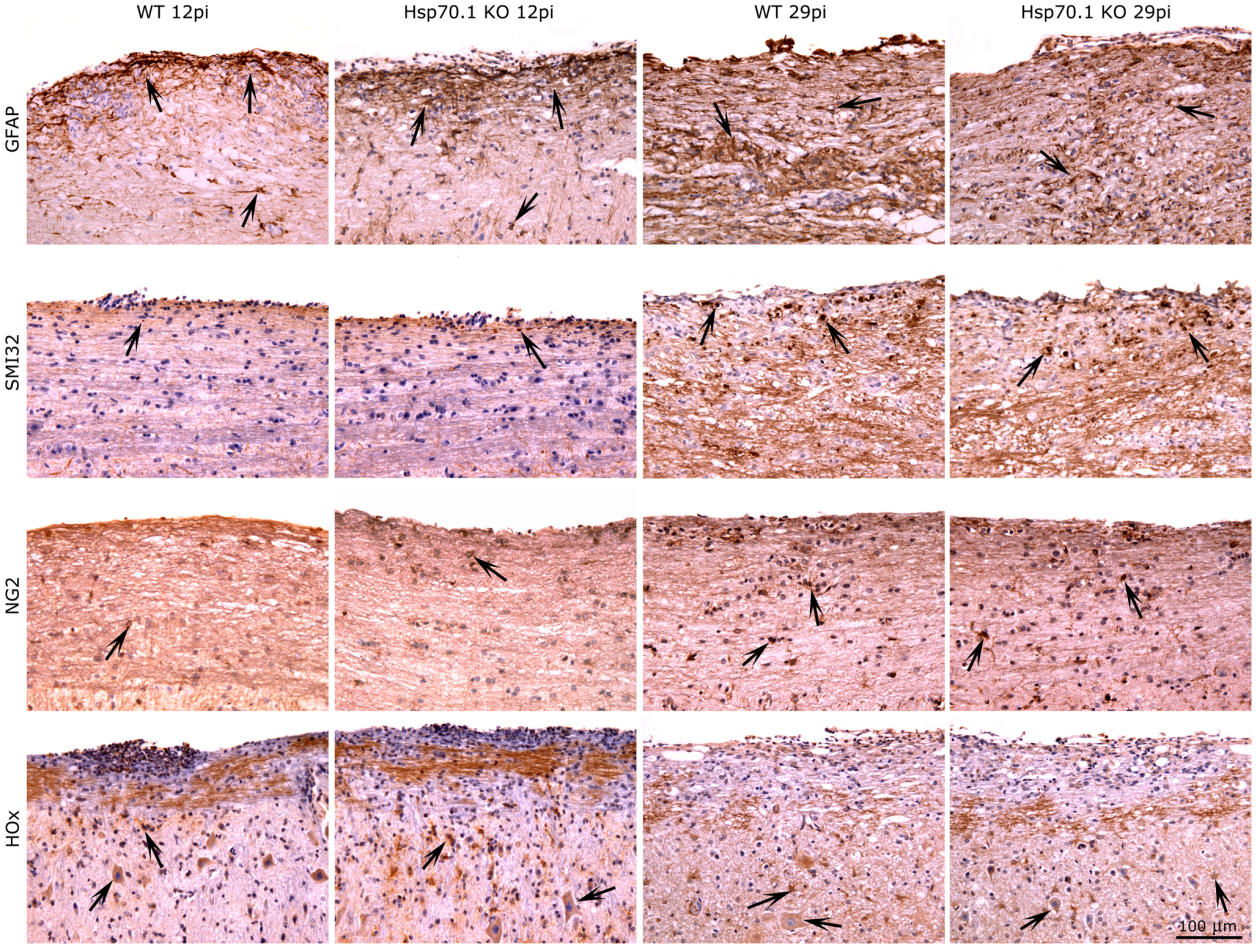
Immunoshitochemical staining for GFAP, SMI32, NG2 and HOx of the spinal cord of Hsp70.1 KO and WT mice. Immunohistochemical staining for GFAP (astrocytes), SMI32 (axonal damage) and NG2 (oligodendrocyte progenitor cells) was performed in Hsp70.1 KO and WT mice at days 12 p.i. or 29 p.i. Hsp70.1 KO mice showed higher expression of oxidative stress marker HOx than WT mice at day 12 p.i. Arrows indicate positive stained cells.

Immunohistochemical studies using oxidative stress markers revealed that the Hsp70.1 KO mice showed a significantly higher expression of HOx (HOx: 66.34±3.30 *vs*. 38.50±3.54, p = 0.015) at day 12 p.i., although the levels of MDA, NITT and iNOS remained similar in both groups. In contrast, no differences were observed in the numbers of infiltrating cells (CD45+ and CD3+ cells), macrophages/microglia (LEA+ cells) and astrocytes (GFAP+ cells) or in the axonal damage (SMI32+ cells) and remyelination (NG2+ cells) in the spinal cord of the Hsp70.1 KO and WT mice (Fig. 4).

### *In vitro* model of CNS inflammation

**Figure 5:**
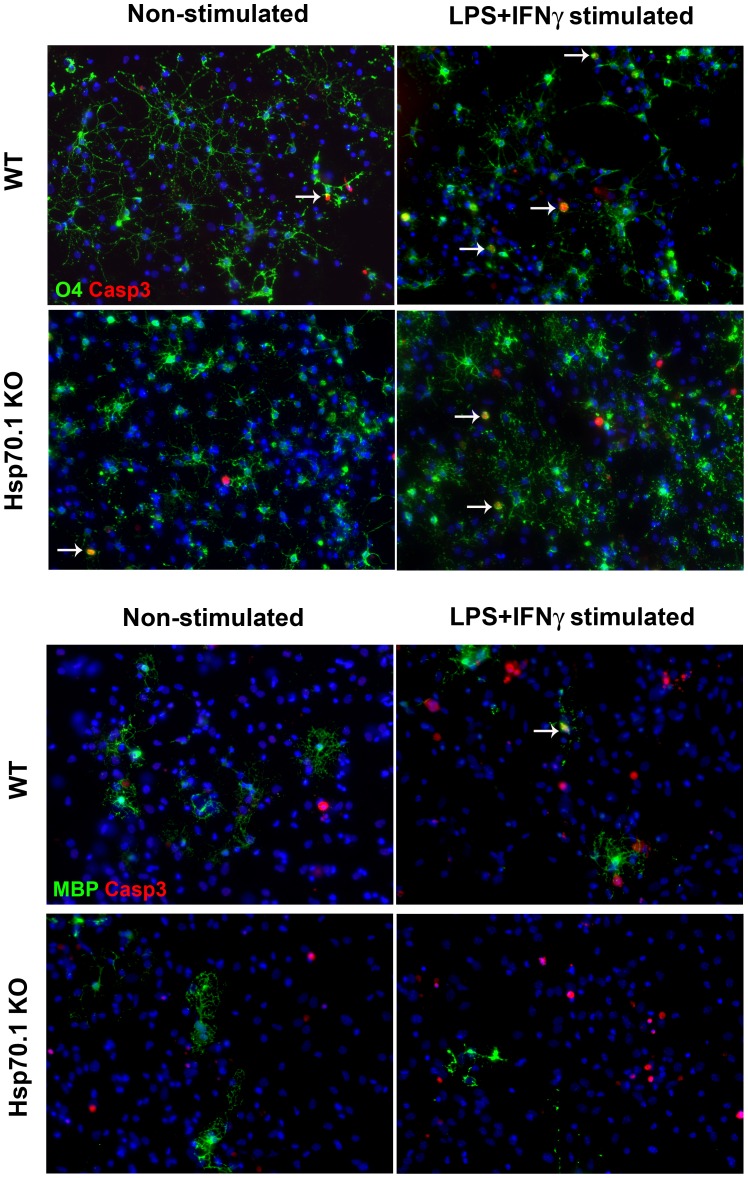
Apoptotic induction in CNS cell cultures. Apoptotic immunocytochemical detection in oligodendrocyte precursor cells (OPCs, O4+ cells) and mature oligodendrocytes (MBP+ cells) in mixed CNS cell cultures of WT and Hsp70.1 KO mice under non-stimulated (left column) and LPS plus IFN-γ-stimulated (right column) culture conditions. A. Detection of apoptotic OPCs cells (O4+Casp3+ cells). The rows indicate the O4+Casp3+ cells. B. Detection of apoptotic mature oligodendrocytes (MBP+Casp3+ cells).

A mixed culture of CNS cells consisting of 40.5% of neurons, 25.3% of oligodendroglial cells [20.9% O4+ cells (OPCs) and 4.4% MBP+ cells (mature oligodendrocytes)] and 34.2% astrocytes was obtained from the Hsp70.1 KO or WT mice, as previously described in the Material and Methods section. No intrinsic microglia (isolectin B4+ cells) was detected in the CNS cell cultures (data not shown). To determine the optimal conditions to induce apoptosis after 24 or 48 h of incubation, the culture medium was replaced with fresh medium containing a different number of microglial cells in combination with increased concentrations of LPS and IFN-γ. We established that after 24 h of incubation 16,000 microglial cells activated with 100 ng/ml LPS and 100 ng/ml IFN-γ induced apoptosis (Casp3+ cells) while those cultures containing non-activated microglia hardly showed apoptotic cells (Table 1 and Fig. 5).

**Table 1:** Characterisation of the non-stimulated CNS cell cultures and following an inflammatory stimulus.

		Non-stimulated	LPS+IFN-γ stimulation	Fold change
**% O4+ cells**	**WT**	20.94±0.09	15.87±0.22	0.76±0.01
	**Hsp70.1 KO**	15.09±5.43	10.09±3.23	0.67±0.03
**% MBP+ cells**	**WT**	4.37±0.75	2.85±0.07	0.67±0.13
	**Hsp70.1 KO**	2.93±0.13	1.15±0.97	0.39±0.32
**% Casp3+ cells**	**WT**	1.80±0.63	4.01±1.66	2.51±0.86
	**Hsp70.1 KO**	2.31±0.58	3.57±0.63	1.60±0.33
**% O4+Casp3+ cells**	**WT**	0.51±0.43	2.19± 0.28	6.45±4.96
	**Hsp70.1 KO**	0.97±0.36	1.64± 0.58	1.70±0.13

The percentage of O4+, MBP+ and Casp3+ cells from the total number of cells of non-stimulated or LPS plus IFN-γ-stimulated cultures from wild-type (WT) and Hsp70.1 knock-out (KO) mice after 24 h. Fold change induced by inflammatory stimulus of each population was calculated referred to matched non-stimulated cultures. The results were expressed as the mean±SD.

### Effect of the inflammatory stimulus in the CNS cell cultures

As shown in Table 1, after an inflammatory stimulus we observed a significant increase in the percentage of apoptotic cells (Casp3+ cells) in both Hsp70.1 KO (p<0.001) and WT (p = 0.025) CNS cell cultures. Among the different apoptotic cells, OPCs (O4+Casp3+ cells) were the most affected in both groups. We were not able to detect apoptotic mature oligodendrocytes (MBP+Casp3+ cells) or apoptotic neurons (NeuN+Casp3+ cells) (data not shown). Altogether these data suggest that OPCs are susceptible to the induction of apoptosis upon an inflammatory stimulus.

**Figure 6:**
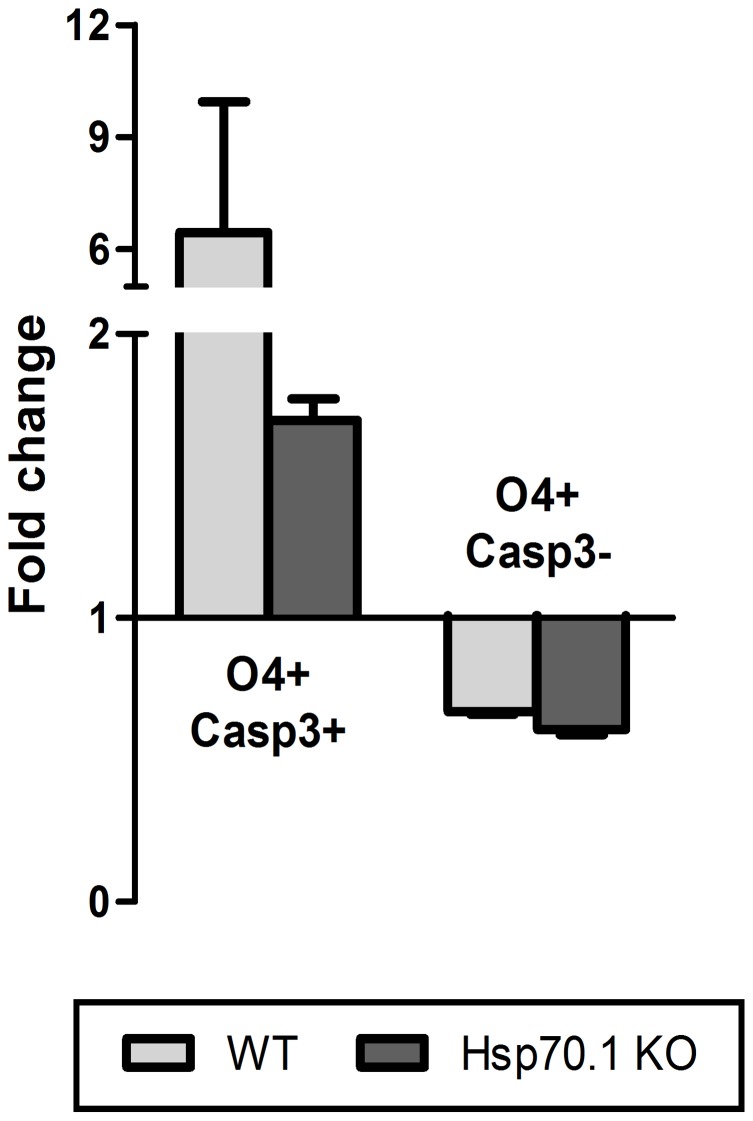
Oligodendrocyte precursor cell susceptibility to apoptotic induction following inflammatory stress. Analysis of Casp3 expression in O4+ cells following an inflammatory stress. The fold change of apoptotic (Casp3+) and viable (Casp3-) O4+ cells was similar in the WT and Hp70.1 KO CNS cell cultures after 24 h of stimulation with LPS plus IFN-γ. The error bars correspond to the SEM.

To investigate the cytoprotective functions of Hsp70.1, we studied the vulnerability of OPCs to the inflammatory stress in Hsp70.1 KO compared with WT CNS cell cultures. When the apoptotic induction was analysed, we did not observe a statistically significant difference in the fold change of apoptotic OPCs (O4+Casp3+) in Hsp70.1 KO respect to WT CNS cell cultures (Table 1, Fig. 6). These data suggest that the absence of Hsp70 did not influence the susceptibility of OPCs (O4+ cells) to inflammation-induced apoptosis.

## Discussion

Several recent studies have described the important role of Hsp70 not only as an essential chaperone but also as an anti-apoptotic mediator and promoter of both innate and adaptive immune responses [11]–[13], [15], [16], [18], [35]–[37]. To study the role of Hsp70 in the pathogenesis of MS, we used a MOG-induced EAE animal model. We observed that Hsp70.1 KO mice were significantly more resistant to EAE development compared with their WT littermates. Nevertheless, the Hsp70.1 KO mice that developed clinical symptoms of EAE showed the same clinical course and histopathological characteristics as the WT mice. Thus, Hsp70.1 appears to be relevant, but not crucial, for the EAE outcome. These results are partially consistent with previous studies performed by Mycko and colleagues, in which all animals developed clinical signs of the disease but the Hsp70.1 KO mice developed a milder form of EAE. Furthermore, these authors also demonstrated that the passive transference of Hsp70.1-deficient lymphocytes with either MOG antigen- or polyclonal restimulation did not induce EAE in C57BL/6 WT mice [38].

Knock-out mouse technology is an important approach used to investigate the function of genes that are not essential for embryogenesis and development in the animal. However, under some circumstances, KO mice develop compensatory mechanisms that overcome the genetic deficiency [39]. One of the most common compensatory mechanisms consists of the up-regulation of genes with the same or similar functions. The inducible form of Hsp70 is encoded by the Hsp70.1 and Hsp70.3 genes, although the Hsp70.1 gene deletion is well known to be sufficient to abrogate inducible Hsp70 activity in mice [40]. As Hsp70 is a relevant protein for homeostatic functions following stressful situations, it would be plausible that Hsp70.1 KO mice would develop some compensatory mechanisms. For this reason, we performed an experiment using siRNA technology to down-regulate the Hsp70.1 protein expression after EAE induction. To achieve this outcome, we first immunised WT mice with the MOG_35–55_ peptide. When the first EAE clinical symptoms were detected, the animals were randomised into two groups and then injected with either Hsp70.1 or non-targeting siRNA. The mice also received a second dose of siRNA on day 14 p.i. Compared with the mice administered with non-targeting siRNAs, mice in which Hsp70.1 expression was down-regulated showed an improved EAE clinical course, and one mouse even remained disease-free. In agreement with these data, we previously demonstrated a baseline-increased expression of the HSPA1A gene in PBMCs from MS patients compared with HDs. In addition, we observed an increase in the protein expression of inducible Hsp70 in T lymphocytes (CD4+ and CD8+) and monocytes from MS patients under basal conditions that may reflect the immunological activation occurring in MS patients [29]. Taken together, these data confirmed and further supported the Hsp70.1 KO mice results and indicate a relevant role of Hsp70.1 in the EAE outcome.

Previous studies have shown that Hsp70 released into the milieu can act as a danger signal or proinflammatory mediator, thereby inducing innate immune responses via the TLR2/4 and CD14 receptors [13], [35], [37]. Furthermore, extracellular Hsp70 can act as an adjuvant that promotes adaptive immune responses against specific antigens. In this context, complexes of Hsp70 and either MOG or MBP were found in MS lesions, and complexes of Hsp70 and either MOG or PLP were present in the CNS of mice with EAE [19], [27], [28]. In contrast to our expectations, splenocytes lacking Hsp70.1 were able to induce an antigen-specific proliferative response, and Hsp70.1 KO mice with EAE also exhibited a higher MOG-specific proliferative response compared with WT mice in the inflammatory and chronic phases of the disease. Nevertheless, the high proliferative response observed in the incident Hsp70.1 KO mice was not related to an increased cytokine production in either the inflammatory or chronic phase of the disease. We only observed a reduction of the IL-2 levels in the Hsp70.1 KO splenocytes compared with the WT cells, probably related with its consumption due to the increased proliferative cell response observed in Hsp70.1 KO mice. We confirmed these results using siRNA technology to abrogate Hsp70.1 protein expression after EAE induction. Otherwise, the differences in the proliferative response did not involve differences in the extension of the inflammatory infiltrate observed in the spinal cords of incident Hsp70.1 KO and WT mice in both the inflammatory and chronic phases of EAE, and the same was observed when siRNAs were used. However, it is remarkable to note that although we observed a higher clinical improvement in the Hsp70.1 siRNA treated mice than in Hsp70.1 KO mice, the differences in splenocyte proliferation were milder using siRNAs. siRNA technology allows to avoid compensatory mechanisms that Hsp70.1 KO mice could have developed. Thus, the clinical effect of Hsp70.1 deficiency would be more potent in mice treated with Hsp70.1 siRNA than in Hsp70.1 KO mice. However the effect of siRNA is transitory and we performed splenocyte proliferation studies in siRNA-treated mice only at the end of the experiment (day 29 p.i.), 15 days after the last siRNA administration. The fact that at this time we were not able to detect down-regulation of Hsp70.1 protein expression could explain the differences in splenocyte proliferation between the two approaches that we used to inhibit the expression of Hsp70.1. Taken together, these data might indicate that only animals that developed an enhanced MOG-response achieved a sufficient level of cytokine production to proper establish an inflammatory environment to develop EAE. Mycko and colleagues reported that Hsp70.1-deficient CD4+ T cells were more susceptible to apoptosis following anti-CD3 stimulation [38], thereby supporting our hypothesis that Hsp70.1 KO mice required a higher number of antigen-specific T cells compared with the WT mice to induce an effective response against myelin antigens.

Classically, HSPs, including Hsp70, have been defined as proteins involved in proper protein folding with cytoprotective functions under stressful conditions [1]–[4], [6]–[8]. However, Hsp70 over-expression in neuronal cell cultures and mouse models of some neurodegenerative diseases have demonstrated a beneficial reduction in abnormal protein aggregation, such as amyloid peptides [41], [42], tau protein [43], huntingtin [44] and α-synuclein fibril formation [45]. To study the function of inducible Hsp70 as a cytoprotective molecule in the CNS under inflammatory conditions occurring in EAE and MS, we established an *in vitro* model consisting of a mixed CNS cell culture in which an inflammatory stimulus was induced. We observed that Hsp70.1 deficient CNS cell cultures did not present a higher vulnerability to the induction of apoptosis in OPCs (O4+ cells) under inflammatory conditions compared with WT CNS cell cultures. Accordingly, when histopathological studies were performed in Hsp70.1-deficient mice that developed EAE, in both Hsp70.1 KO mice and Hsp70.1 siRNA-treated mice, neither the number of NG2+ cells (OPCs) nor the extent of demyelination were affected. In addition, the amount of axonal damage or astrogliosis were comparable to those observed in the WT or non-targeted siRNA-treated mice.

## Conclusions

Our results indicated that although Hsp70.1 could play a role in both the immune response and cytoprotection of CNS cells, in the MOG-induced EAE model, Hsp70.1 seems to play a more relevant role in promoting an effective T cell response against the auto-antigen compared with its role in protecting CNS cells from inflammatory injury. Consequently, specific therapies down-regulating the expression of Hsp70 may be a promising approach to reduce or control the early autoimmune response in MS patients.
